# California State Dental Association

**Published:** 1871-08

**Authors:** 


### Proceedings of Societies.

### CALIFORNIA STATE DENTAL ASSOCIATION.

The second annual meeting of the California State Dental Association, was held at the rooms of the San Francisco Dental Association, San Francisco, June 6th, 7th and 8th, 1871.

The association was called to order at 10:30 A. M., by the President, Dr. C. C. Knowles.

President Knowles delivered his annual address, which was received with applause, and that portion of it relative to the education and elevation of the profession, referred to the Committee on Dental Literature and Education, and address in full to the Publication Committee.

The several Standing Committees asked further time to report. Granted.

Drs. Austin, Younger and Dennis, were appointed to select subjects for discussion.

An amendment to the by laws, laid over from last session, creating an Auditing Committee, was taken up and passed.

Drs. T. Crossett and J. O. Belle, of San Francisco, and A. C. Davenport, of Stockton, were admitted to membership.

The committee appointed for said purpose, reported the following subjects for discussion.

1st.—Filling Teeth.

2nd.—Hemorrhage after Extracting Teeth.

3d.—Treatment of Alveolar Abscess.

4th.—The Cause and Treatment of Loose Teeth.

5th.—Treatment of Exposed Pulp.

6th.—Mechanical Dentistry.

7th.—Dental Pathology.

Adjourned to 1:30 P. M.

### AFTERNOON SESSION.

Called to order at 1:30 P. M. President Knowles in the chair.

Minutes of morning session read and approved.

The subject of "Filling Teeth" was taken up, and thoroughly discussed by nearly every member present, the discussion lasting nearly three hours.

(Ed.—A synopsis, even of the discussion would be too long for your purpose—Pub. Committee.)

Dr. C. Hamilton, of Unionville, Nevada, was admitted to membership.

Adjourned to 8 P. M.

### EVENING SESSION.

Called to order at 8 P. M. President Knowles in the chair.

An application was received from a gentleman claiming to be a member of the profession, and after being duly examined, permission was given to the applicant to withdraw said application. The members, however, were unanimous in the opinion and determination to admit none to membership but those who could come well recommended as to professional honor, good moral character, etc., etc., as it is desirous to make our association equal to any in the United States, so far as the honor, professional and otherwise of its members are concerned.

Subject of Filling Teeth again discussed.

Dr Austin read an essay on the "Pathology and Treatment of Alveolar Abscess," which was of much interest to those present, and contained very much that was endorsed by the association.

Adjourned to 10 A. M., to-morrow.

### SECOND DAY—MORNING SESSION.

Met at 10 A. M. President Knowles in the chair.

Roll called, and minutes of last session read and approved.

Dr. Sichel, exhibited specimens of Hyatt & Perkins' base for artificial dentures.

Dr. F. M. Shields, of Sacramento, and L. G. Yates of Centreville, were admitted to membership.

Dr. S. W. Dennis, read an essay on the "Causes of the Degeneracy of the Teeth, especially among Children," by S. P. Cutler, M. D., D. D. S., of New Orleans, La.

The association tendered to Dr. Cutler, a vote of thanks for his kindness in furnishing us the very able paper, and made him an honorary member.

The subject matter of Dr. Cutler's essay was discussed at length.

Adjourned to 2 P. M.

AFTERNOON SESSION.

Called to order at 2 P. M.,.by President Knowles.

Roll called, and minutes of morning session read and approved.

Drs. Younger, Dutch, and Crossett, were appointed a committee to draft resolutions appropriate to the memory of Drs. F. A. Park, and H. J. Paine, deceased since our last annual session.

The subject of "Hemorrhage after Extracting Teeth," taken up and thoroughly discussed.

Dr. Sichel, favored mechanical pressure to suppress hemorrhage when possible.

Dr. Bush related a case where profuse hemorrhage followed the extracting of a deciduous superior molar. Failing to arrest it with the usual remedies, finally succeeded with *ice*, applying direct to the socket and surrounding gums.

"Treatment of Alveolar Abscess," was next discussed.

Dr. Younger claimed that not five per cent. of cases treated by him were unsuccessful. His favorite remedy was saturated solution of iodine, to be thoroughly pumped into the sac, and continued until cure is effected.

Adjourned to 8 P. M.

EVENING SESSION.

Met at 8 P. M. President Knowles in the chair.

Officers all present, excepting J. Ball, librarian.

Minutes of afternoon session read and approved.

Drs. J. A. Woodward, of San Francisco, and N. Klein, of San Jose, were admitted to membership.

Hour of special order having arrived, proceeded to election of officers for ensuing year, with following result:

*President*—S. W. Dennis, of San Francisco.

*Vice Presidents*—H. H. Pierson, Sacramento; J. J. Menefee, San Jose; T. Crossett, San Francisco.

*Corresponding Secretary*—H. E. Knox, San Francisco.

*Recording Secretary*—H. J. Plomteaux, Woodland.

*Assistant Recording Secretary*—J. N. Myers, Stockton.

*Treasurer*—C. C. Knowles, San Francisco.

*Librarian*—A. C. Davenport, Stockton.

Dr. Knowles retired from the chair, and in appropriate remarks introduced the President elect, who with the other officers assumed their respective stations, and at once entered upon the discharge of their duties.

Drs. J. J. Birge, Wm. Dutch, H. H. Pierson, S. W. Dennis and W. W. Light, were elected delegates to the National Dental Association.

Adjourned to 10 A. M., to-morrow.

After the election, the association went in a body to partake of the bountiful supply of the luxuries of life, prepared under the auspices of the San Francisco Dental Association, where the sentiment seemed general that the meeting was a success.

THIRD DAY—MORNING SESSION.

Association met at 10 A. M , and was called to order by the President, Dr. S. W. Dennis.

Roll called, officers all present.

Minutes of last session read and approved.

Committee on Mechanical Dentistry reported that they had received no information as to the merits or demerits of the Rose Pearl work, presented by Dr. Smith at our last annual session, consequently had nothing further to report concerning it.

Dr. S. W. Dennis, in behalf of the Committee on Dental

Literature and Education, made a very interesting report. Referred to Publishing Committee.

Several resolutions of amendments to the Constitution, were read and laid over under the rule, until next annual session.

Dr. Belle, offered a series of resolutions relative to certificates of membership, which was referred to Drs. Knowles, Birge, and Menefee.

Dr. Knox, offered the following, which was adopted :

*Resolved*, That our delegates to the American Dental Association be, and are hereby instructed to use all their influence, that the session of 1872, of said association, be held in San Francisco.

Notice was given, that previous to the commencement of the afternoon session, Dr. Knox would illustrate the use of the rubber dam.

The President was authorized to fill any vacancy that may occur from death during the coming year, in any of the Standing Committees, by appointment through the Recording Secretary.

Adjourned to 1:30 P. M

THIRD DAY—AFTERNOON SESSION.

Called to order at 2:30 P. M. President Dennis, in the chair.

Minutes of morning session read and approved.

The following preamble and resolutions were unanimously adopted :

IN MEMORIAM.

WHEREAS, It has pleased Almighty God in His infinite wisdom, to remove from our midst our late brothers, Drs. F. A. Park, and H. J. Paine ; therefore,

*Resolved*, That in the death of brothers Park and Paine, the dental profession has been deprived of two of its most active, efficient, and honorable members, and the community of just and upright citizens.

*Resolved*, That we, the members of the California State Dental Association, deplore the grievous loss which their families have sustained, and cordially sympathize with them in their great bereavement.

*Resolved*, That the Corresponding Secretary be instructed to forward a copy of these resolutions to Mrs. F. A. Park, and Mrs. H. J. Paine, and that a copy be spread upon the minutes of the association.

Dr. W. J. YOUNGER,
Dr. WM. DUTCH,
Dr. T. CROSSETT.

The committee to whom that part of President Knowles' annual address, relative to dental education, etc., was referred, reported a series of resolutions heartily approving his suggestions, and recommended that stringent rules should be adopted to prevent the admission of members to our society, who have not a well established reputation for scientific attainments. Also, recommending that members do not fellowship professionally with those not possessing the above qualifications, etc. Report accepted.

Committee on Publication, reported estimates of cost of publishing monthly periodicals, size of the "*Dental Office and Laboratory*," and size of the "*Missouri Dental Journal.*"

Dr. Knowles offered the following:

*Resolved*, That it is expedient that a dental periodical be published under the supervision of this association.

Referred to Committee on Publication, with instructions to ascertain the facts relative to aid from the members, probable number of advertisements to be secured, etc.

Dr. Dennis read a letter from Dr. E. A. Bogue, soliciting aid to prepare a suitable testimonial for Dr. S. C. Barnum, inventor of the rubber dam.

Dr. Dennis made a pressing appeal in behalf of said inventor, and the matter was referred to a committee consisting of Drs. Knowles, Younger, and Dutch, to take into consideration the propriety of making some substantial recognition to Dr. Barnum for his valuable invention, and our delegates to the American Dental Association, were instructed to use their influence for some general plan, whereby a suitable "national testimonial" can be secured for Dr. Barnum.

The President appointed the Standing Committees for ensuing year, as follows:

*Committee of Arrangements.*—Drs. Birge, Davenport, Shields, Yates and Dutch.

*On Publication* —Drs. Dennis, Plomteaux, (ex officio) Knowles, Younger and Bunnell.

*On Scientific Investigation.*—1*st Section—Operative Dentistry*—Drs. Dutch, Menefee and Pierson.

2*nd Section—Dental Chemistry.*—Drs. Light, Sichel and Belle.

*On Dental Pathology and Surgery.*—Drs. Austin, Crossett, Prather, Kingsbury and Klein.

*On Mechanical Dentistry.*—Drs. Woodward, Bush, Myers, Cool and Hamilton.

*On Dental Literature and Education.*—Drs. Knox, Younger, Crossett, Eaton and Knowles.

*Auditing Committee.*—Drs. Menefee, Light and Myers.

San Francisco was chosen as the place for next annual session.

On recommendation of Dr. Dennis, Professors J. H McQuillen, Homer Judd, and J. Taft, were declared honorary members.

The President assigned subjects for thesis to be read at next annual session, as follows:

Dental Therapeutics—Dr. Knox.

On the Causes of Degeneracy of the Teeth, especially among Children—Dr. Birge.

On the Causes of Irregularity of the Teeth, and best method of correcting the same, to Dr. Knowles.

On the Pathology and treatment of Alveolar Abscess, to Dr. Menefee.

On Dentistry, its history, present status, claims and relations, to Dr. Plomteaux.

On Professional Ethics—Dr. Bunnell.

On the Causes and treatment of Loose Teeth, to Dr. Younger.

On motion, adjourned to meet in San Francisco, second Tuesday in June, 1872, at 10 A. M.

